# Sexual Dimorphism for Coping Styles Complements Traditional Methods for Sex Determination in a Multivariety Endangered Hen Breed

**DOI:** 10.3390/ani9121165

**Published:** 2019-12-17

**Authors:** Carlos Iglesias Pastrana, Francisco Javier Navas González, Carmen Marín Navas, Ander Arando Arbulu, Antonio González Ariza, José Manuel León Jurado, María Gabriela Pizarro Inostroza, Maria Esperanza Camacho Vallejo

**Affiliations:** 1 Department of Genetics, Faculty of Veterinary Sciences, University of Córdoba, 14071 Córdoba, Spain; carlos3b06@hotmail.com (C.I.P.); carmen95_mn@hotmail.com (C.M.N.); anderarando@hotmail.com (A.A.A.); angoarvet@outlook.es (A.G.A.); 2 Centro Agropecuario Provincial de Córdoba, Diputación Provincial de Córdoba, 14071 Córdoba, Spain; jomalejur@yahoo.es; 3 Animal Breeding Consulting, SL, 14014 Córdoba, Spain; kalufour@yahoo.es; 4 Instituto de Investigación y Formación Agraria y Pesquera (IFAPA), Alameda del Obispo, 14004 Córdoba, Spain; mariae.camacho@juntadeandalucia.es

**Keywords:** morphometric measurements, behavior, preincubation, postincubation, sexing tests, autochthonous breed

## Abstract

**Simple Summary:**

Early determination of sex of poultry specimens plays a major role in the design and implementation of conservation programs for endangered avian species. This information can be used to tailor noninvasive early specific models to determine sex, fitting the characteristics of local poultry populations, as traditional methods may not be effective given the implicit diversity of local breeds and their varieties or strains. The English method, down feather coloration, wing fan, and behavior/coping styles displayed by the individuals can be used to accurately sort animals according to their sex, regardless of the variety of the individuals.

**Abstract:**

Sex determination is key to designing endangered poultry population conservation and breeding programs when sex distribution departs from Hardy–Weinberg equilibrium. A total of 112 Utrerana chickens (28 per variety, partridge, black, white, and franciscan) were selected for hatching day sexing. Sex assignation was performed through 10 methods. Three sex assignment criteria comprised criteria found in literature, opposite criteria to that in the literature, and composite criteria combining methods reporting the highest predictive success from the previous ones. This study aims to determine which method combinations may more successfully determine sex across the four varieties of Utrerana endangered hen breed to tailor noninvasive early specific models to determine sex in local chicken populations. Although the explanatory power of the three assignation criteria is equal (75%), assignation criteria 2 resulted to be the most efficient as it correctly assigns males more frequently. Only methods 3 (English method), 5 (general down feathers coloration), 7 (wing fan), and 10 (behavior/coping styles) reported significant differences regardless of the variety, hence, are appropriate for early sexing. Sex confirmation was performed at 1.5 months old. Identifying sex proportions enhances genetic management tasks in endangered populations, complementing more standardized techniques, which may result inefficient given the implicit diversity found in local populations.

## 1. Introduction

The poultry industry is, in terms of the production of local breeds in a sustainable way, a currently booming sector [1,2,3]. The pressure to meet the growing demands for poultry products (meat and eggs) has led to the implementation and improvement of new techniques and work tools to improve productivity, such as the sexing of chickens in farms [4], as early in the life of the individuals as possible. This allows sex segregation and the withdrawal of those individuals without zootechnical interest from the production cycle (e.g., males in egg layer farms), with the consequent economic and logistical benefit that this implies for the poultry industry.

Apart from the economic interests from food related industries, the knowledge of the relative distribution of sex in fowl populations is considered to be of a special relevance in the case of studies following an evolutionary, ecological, and behavioral perspective. In this context, the determination of the sex of individuals is a key element in the design and planning of conservation and breeding programs [5,6,7] for threatened or endangered species or breeds for which sex distribution may be biased or what is the same, whose characteristics may depart from a Hardy–Weinberg equilibrium [8].

Vent sexing, venting, or Japanese examination is the most used traditional method of determining sex in one-day-old chickens, along with the sexing from the primary and secondary wing feathers [9]. Qualified and trained professionals are able to appreciate the subtle morphological differences that exist between the genital eminences and local folds of males and females, by visual examination of the cloaca of the animal in the first hours after breaking the shell (first 12–26 h) and before the chicks start eating [10,11]. However, this method is complex and reaching an efficient enough sexing performance of operators requires investing in time, training, and economic resources.

The advances in genetics and reproduction have enabled the obtention, through guided crosses, of offspring for whom the sex of the individuals can be determined based on specific phenotypic characters at the time of birth or a few days later (auto-sexing and semi auto sexing), what simplifies the process. These methods rely on the growth rate of the feathers of the outer edge of the wing (primary feathers and secondary feathers or coverts) [9,10,12,13] or the color patterns in the first days after hatching [14]. This methodology is not usually used in commercial practice, since its value is debatable given the time needed to be able to perform chicken sexing after their birth as differences in appearance are commonly emphasized as the animals grow [11]. Other examples of noninvasive sexing techniques in chicks are the monitoring of the degree of opacity of the eggs during their incubation (with those eggs displaying a higher opacity resulting in male chickens) [15] and the identification of possible behavioral differences depending on the sex [16,17,18,19].

On the other hand, there are invasive techniques to determine the sex of chickens, such as the Kizawa method or proctoscope sexing and molecular sexing. In the first, the gonads can be seen through the intestinal wall, using a cannula equipped with an optical augmentation system and a light bulb that is introduced through the rectum of the animal [10,11]. This method has been used successfully for day-old chicks and has the advantage of requiring less training and skill than the Japanese method. The molecular method, on the other hand, implies the identification of chromosomes by karyotype or the biochemical characterization of the genetic information of the animal, thus reserving this methodology of sexing for poultry strains of high economic interest [20,21].

The growing concern for animal welfare has placed the industrial practice of eliminating male chickens in laying strains as one of the greatest ethical problems in modern poultry [22]. In this context, the use of accurate and reliable techniques for sex determination of endangered fowls is of special importance in breeding programs, especially in captive ones displaying monogamous behavior, with problems or absence of copulation, and with a low hatching rate [23]. In view of this situation, numerous research works are being carried out to develop a technique of sexing of chickens during the first days of embryonic development, since it has not yet developed sensitivity, and hence, there is no suffering [24].

The first in ovo sexing techniques available for industrial application are invasive in nature, as they require sampling for the production of a hormonal or genetic profile, which in turn ends up affecting the posterior hatchability of the egg. In addition, its application is limited only to the period of sexual differentiation of the embryo [25,26].

New tendencies of in ovo sexing systems focus on the lack of contact required (noninvasive methods) and early determination (as they allow the determination of sex on day 3.5 of incubation). One of the most remarkable discoveries makes use of Raman spectroscopy to analyze the spectrum of the circulating blood of the extraembryonic vessels, with a 90% accuracy. This technique is in its early stages of development and refinement before it can be implemented at a commercial scale in the incubation plants [25].

Out of this commercial scale, endangered fowl populations may benefit from early sex confirmation, even if eliminating one sex or the other is not the aim, but to implement and design better population management strategies to preserve genetic diversity, when sex distribution across the population may be biased. Contextually, local fowl populations have additional issues regarding the fact that standardized methods may not be appropriate to determine sex, as sex may be conditioned by marked inner external features typical from each breed or variety [27].

Morphological development of the gonads appears to have been conserved through evolution across animal groups. However, in vertebrates, sex has been suggested to be determined by a surprising variety of mechanisms including chromosomally (CSD) or environmentally linked ones (temperature dependent sex determining (TSD) mechanisms). Therefore, some authors concluded that although the basic genetic pathway controlling the morphological differentiation of the gonads appears to have been conserved, the genetic mechanisms triggering sex determination may involve an important diversity of signs [28].

Sexual dimorphism comprises the differences between males and females of the same species, such as in color, shape, size, structure, behavior, or cognition that can be attributed to the inheritance of sexual genetic material [29]. An important finding to have emerged from poultry studies is that sexual dimorphism at the molecular level can occur well prior to sexual dimorphism at the morphological level. Genes such as *DMRT1*, *ASW*, and *FET1*, for example, all show sexually dimorphic expression as early as day 3.5, well prior to the histological onset of gonadal sex differentiation at day 6.5 [30]. The W chromosome represents less than 2% of the chicken genome and contains only a few coding sequences, in contrast with the Z chromosome, which mainly includes the genes influencing feather color and growth patterns [31,32]. In this context, criss-cross inheritance magnitude of sex-linked traits is grossly determined by the crossing direction and the presence of certain dominant alleles in male or female genotypes [14,33].

In this regards, sexual dimorphism in avian species has been reported to affect several secondary phenotypical traits that can be noticeable even from newly laid eggs. These secondary phenotypical traits range from egg size [34,35], feather color, morphology and distribution [36,37], appendicular skeleton dimensions (tarso-metatarsus length) [38], head length and size [39,40], tail inclination or lateralization [41], or even behavioral patterns or cognitive processes [39,42].

When we consider sex determination through secondary external features, this inner diversity related to sex may join the implicit diversity present in the gene pool of genetically closer populations such as breeds or varieties [27]. This specific context makes sex determination more challenging, given the lack of specific protocols to determine sex for each population, but more relevant as certain better productive features may be linked to either one or the other sex in specific local breed or variety populations [43,44,45], Then, the choice of one method or another will ultimately depend on the degree of accuracy or certainty desired, the expected performance of the technique (number of individuals sexed/h), the availability of qualified and experienced professionals, the possible effects of sex selection for ‘unwanted’ or less profitable animals, and economic and financial conditions [46,47].

For these reasons, the aim of this study is to determine which method combinations may more successfully determine sex across the four varieties of Utrerana endangered hen breed. This information will be processed to tailor noninvasive early specific models to determine sex in local chicken populations. Identifying sex proportions in endangered populations can contribute to the improvement and progress of the genetic management tasks carried out in such populations, as an alternative or complement to more standardized techniques, which may have been proved at a commercial scale, but which may result inefficient given the implicit diversity found in related local populations.

## 2. Materials and Methods

### 2.1. Institutional Animal Care and Use Committee Statement

The center involved in the study followed specific codes of good practices and therefore, the animals received humane care in compliance with the national guide for the care and use of laboratory and farm animals in research. The study was conducted in accordance with the Declaration of Helsinki. The Spanish Ministry of Economy and Competitivity through the Royal Decree Law 53/2013 and its credited entity the Ethics Committee of Animal Experimentation from the University of Córdoba permitted the application of the protocols present in this study as cited in the 5th section of its 2nd article, as the animals assessed were used for credited zootechnical use. This national Decree follows the European Union Directive 2010/63/UE, from 22 September 2010.

### 2.2. Sample Size and Background

**Figure 1:**
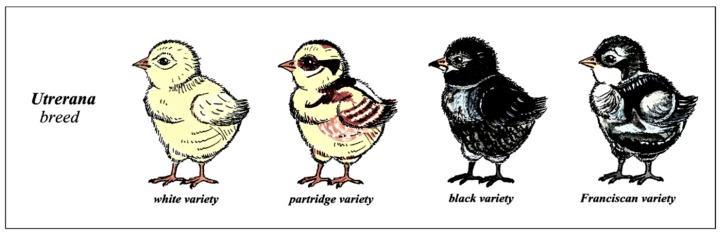
Depiction of Utrerana breed one-day-old chicken varieties.

The experiment was carried out in a public hatchery located at the Agropecuary Provincial Centre of Diputación of Córdoba, Spain (Plus code: W77Q+MF El Levigar/37°54′50.9″ N 4°42′40.4″ W). The eggs used in the study were obtained from a total of 68 autochthonous Utrerana hens at 70 weeks of age. Total hatchability and fecundity for each of the variables tested is reported in Camacho Vallejo, et al. [48]. A total of 1500 eggs were incubated out of which 1261 finally hatched. A random sample of 112 chickens (28 of each variety; black, white, franciscan, and partridge, Figure 1) was selected for sexing at the end of incubation period, on hatching day, as samples around 100 individuals have been reported in literature to report 95% of accuracy in sexing in local poultry species [49]. All eggs were randomly allocated into replicate groups (1 and 2). Eggs from each replicate were collected at the same day and were evaluated on day 0 (for egg maximum width and length parameter), on day 1 or the day that they hatched and a week after day 1), on the same row of breeder flock. The breeder flock consisted of 12 male (four per variety; black, white, franciscan, and partridge with tested fertility) and 68 Utrerana females (17 of each variety; black, white, franciscan, and partridge) at the time of egg collection. Matings were performed between individuals of the same variety.

The flock, from which the eggs were collected, was reared in individual cages (50 × 62 × 41 cm) following Council Directive 1999/74/EC of 19 July 1999, laying down minimum standards for the protection of laying hens at the Agropecuary Provincial Centre of Diputación of Córdoba (Spain), for 6 months (January to June 2018). All the animals were fed on the same commercial feed (15.2% crude protein, 4.1% calcium, 0.66% available phosphorus) for the whole experimental period. Feed and water were available ad libitum. All the fowls were reared according to the regulations of the European Union (2010/63/EU) in their transposition to the Spanish law (RD 53/2013). Further information regarding the rearing system used can be consulted in González Ariza, et al. [50].

The eggs were sanitized and stored at 17–18 °C and 85% Relative Humidity (RH) for four days. Eggs were numbered and information regarding sire, dam, day of laying, variety of parents was collected. Maximum widths and lengths of each egg were measured following a Vernier scale (±0.01 mm) (Electro DH M 60.205, Barcelona, Spain) and weighed individually on an electronic scale with ±0.01 g precision before setting. The eggs were placed at separate incubating platters to be able to trace the animals after birth and incubated in an incubator (Masalles 25 L HLC model with capacity for 180 eggs; Europe SL, Sant Cugat del Vallés, Barcelona, Spain) at 37.2 °C and 55% RH for 18 days. The eggs were turned every hour in an angle of about 45° for 18 days. Eggs were checked for fertility at day 8 to remove eggs for which embryos had not developed. On the 18th day of incubation, all eggs were candled, and fertile eggs placed in a separate chamber in the hatchery cabinet maintained at 36.7 °C and 60% RH until hatching.

### 2.3. Chicken Handling for Examinations

On day 1 and week 1, to make examination easier, the neck of the chicken was held between the index and middle fingers of the left hand, with the head of the chicken down. The abdominal wall was pressed gently with the thumb of the left hand to evert the cloaca and to cause defecation. The surface of the vent was cleaned of urine and feces with a piece of soft tissue paper. The regions to be observed then were manipulated with the index finger and the thumb of the right hand to obtain the optimum view.

### 2.4. Sex Assignation Methods

Sex assignation was performed by three sexers through 10 methods and animals were classified following a dichotomous scale to register the dependent variable of sex considering two levels, male or female (Supplementary Tables S1 and S2).

### 2.5. Sex Assignation Criteria and Method Definition

**Figure 2:**
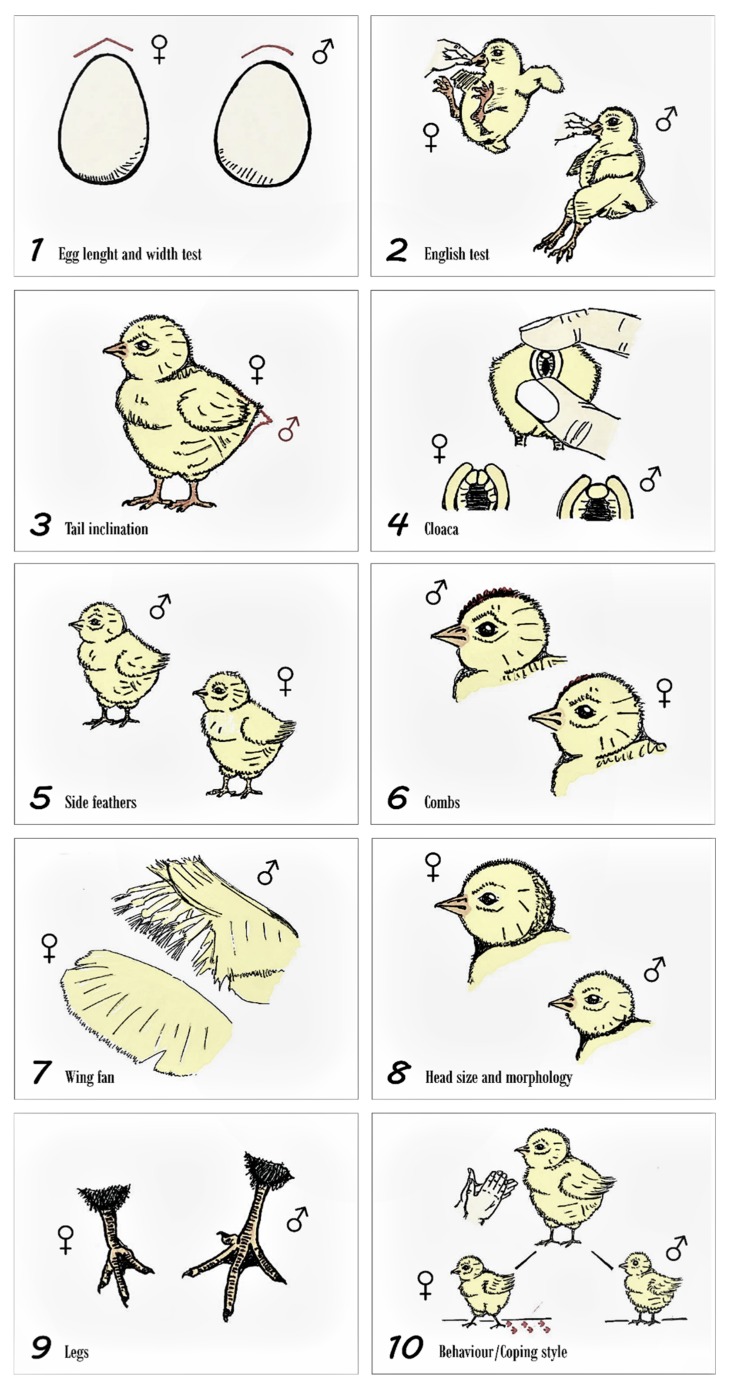
Sex assignment methods and criteria described in literature (Utrerana white variety was used as a reference).

The methods used were previously described by Manuel Llanos Company [11] and are depicted in Figure 2. To check the traditionally proposed methodology for sexing was valid across the different varieties of the Utrerana hen, whether any method could complement the results obtained by the ones traditionally used for this purpose, and to evaluate possible combination strategies that may successfully classify chicken sex more efficiently, we used three possibilities for sex assignation criteria. To this aim, we assessed which sex assignation criteria possibility was able to explain a greater percentage of the existing variability in the sex of the different varieties of Utrerana chickens from one-day-old to 1 month old. First assignation possibility used the criteria previously described in literature to determine whether a chick was a female or a male for each of the 10 methods, second assignation possibility assessed the opposite criteria to that reported in bibliography to assign sex for each of the same 11 methods aiming at addressing possible incongruencies in sex assignation for the four varieties of the Utrerana breed. Third assignation possibility presented a conjugation of the criteria for the 11 methods in a way that it combined those criteria reporting a greater explicative power at sex assignation criteria 1 and 2, with the purpose of maximizing the efficiency of the methods to assign sex correctly across varieties.

The determinants for sex used as a reference in our study, are described below:

(a) Method 1. Egg dimorphism: studied as egg length (1.1) and egg width (1.2). Long, pointed eggs for females and, flat and wide eggs for males. In addition, the date of laying, the identification number of the egg, the possible sex predicted, and the hatching date were recorded. To state the limits to consider an egg long or flat and wide or broad we computed the median of the sizes (sample was not normally distributed *p* > 0.05), to set over and below the median categories. Method 2. English method: holding the chick by the skin of the neck or holding the beak between two fingers, the animal is suspended for 5 s to observe the reaction and posture that it acquires. If the chick kicks, it will be considered a female; if the animal remains motionless, it will be considered a male.
(b) Method 3. Tail Inclination: if the direction of the tail feathers is towards the ground, the individual will be taken as a female. Instead, if the tail is straight, it will be taken as a male.
(c) Method 4. Japanese method or cloaca examination: the basis for this method is the appreciation of morphological differences between the genital eminences and cloacal folds of males and females in newborn chicks. Once the cloaca is externalized applying light pressure with the fingers, attention is paid to the central and ventral part of it. If two small lumps are observed, it will be considered a female. If only one bulge is observed, it will be considered a male.
(d) Method 5. General coloring of down feathers: if the color of down feathers of the sides of the chicken is heterogeneous, it will be considered to be female. If the coloration is homogeneous, it will be taken as a male.
(e) Method 6. Development and changes in the combs from the month and a half onwards: a little developed comb could be characteristic of a female. A higher degree of development of this secondary character could be indicative of the chick being male. Noticeable from 4 to 6 weeks onwards, hence discarded as a sexing method and rather used as a sex confirmation method.
(f) Method 7. Fan-shaped wings and general wing metrics determination: if all of the feathers of each wing are arranged in a well-defined fan shape, this characteristic is considered as specific to the female sex. On the other hand, if the feathers that comprise each wing are at different levels of growth and development, the chick is considered to be male.
(g) Method 8. Body size and head morphology: it seems that males are larger than females within only a few days of life; in addition, his head is usually somewhat smaller and rounded. In females, the morphology of the head is considered to be angled and of greater size than in males. To state the limits to consider a chick big or small we computed the median of the sizes (sample was not normally distributed *p* > 0.05), to set over and below the median categories.
(h) Method 9. Leg length: long legs are considered to be characteristics of male chickens. Short legs will be attributed to female chickens. To state the limits to consider legs long or short, we computed the median of the sizes (sample was not normally distributed *p* > 0.05), to set over and below the median categories. We considered the complete leg, not only the shanks.
(i) Method 10. Behavior/coping styles or slap technique: hands are clapped at a prudent distance of 20 cm from the animal. This technique is applied individually for each chick in an isolated place, apart from the rest of chicks. Two different reactions can be observed: freezing (male) and fleeing or attempting to escape (female).

### 2.6. Sex Confirmation

**Figure 3:**
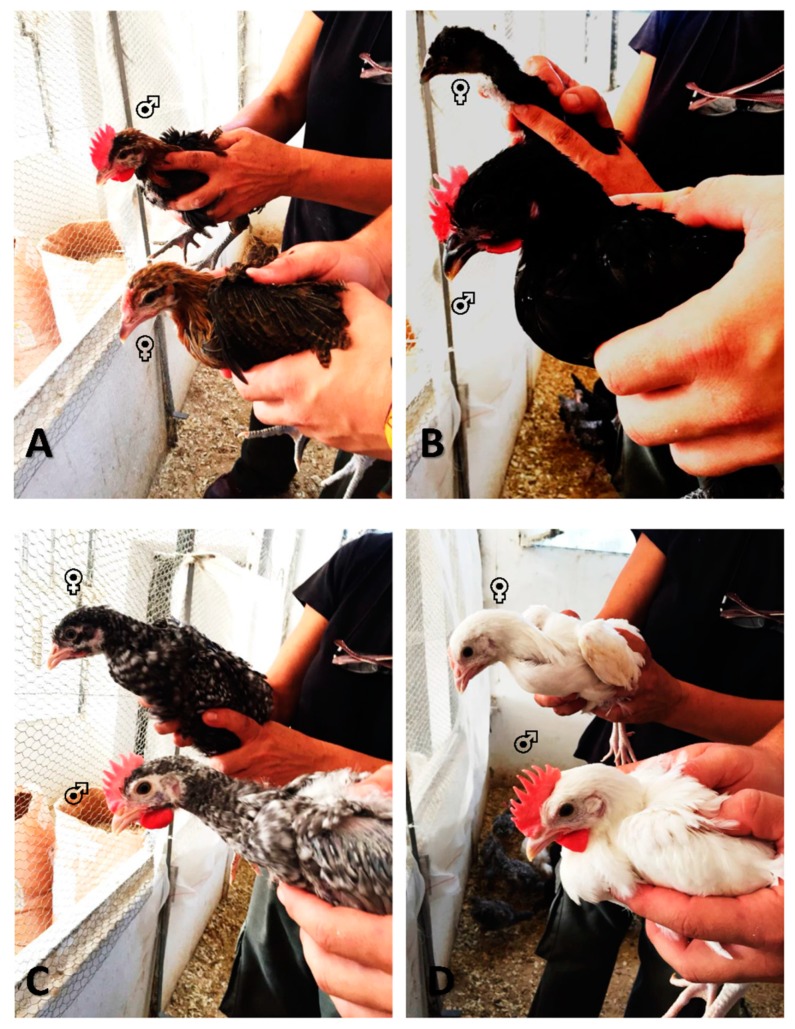
Sex dimorphism across Utrerana hen breed varieties at age 1.5 months with (**A**) showing a Utrerana partridge variety cockerel and pullet, (**B**) a Utrerana black variety cockerel and pullet, (**C**) a Utrerana franciscan variety cockerel and pullet, and (**D**) a Utrerana white variety cockerel and pullet.

Utrerana pullets and cockerels develop secondary sexual features at 6 weeks of age as shown in Figure 3, hence the real sex of the animals could be confirmed.

### 2.7. Sexer Reliability Testing

Intraclass correlation coefficient (ICC), was used for the assessment of absolute agreement and consistency or reproducibility of quantitative measurements made by different observers measuring the same quantity. It describes how strongly units in the same group resemble each other. At this point, defining agreement in terms of consistency or in terms of absolute agreement is compulsory. If we work with a one-way model, only measures of absolute agreement are available, as consistency measures are not defined. However, for two-way models, the default is to produce measures of consistency. The difference between consistency and absolute agreement measures is defined in terms of how the systematic variability due to raters or measures is treated. If that variability is considered irrelevant, it is not included in the denominator of the estimated ICCs, and measures of consistency are produced. If systematic differences among levels of ratings are considered relevant, rater variability contributes to the denominators of the ICC estimates, and measures of absolute agreement are produced [51]. As there were consistent raters for all ratees and a random sample of raters, we used a “Two-Way Random” model. This ICC assumes that the variance of the raters is only adding noise to the estimate of the ratees, and that mean rater error = 0. Or in other words, while a particular rater might rate Ratee 1 high and Ratee 2 low, it should all even out across many raters. It assumes a random effects model for raters, but it explicitly models this effect. This statistic models both an effect of rater and of ratee and assumes both are drawn randomly from larger populations.

ICC relies on the application of a multiple paired Cohen’s κ test, that was run to test for interobserver reliability and determine if there was agreement between three sexers’ judgements on the sexing of 112 chickens across the 10 methods used for sexing. Cicchetti [52] interpreted ICC for clinical tests as less than 0.4 (low repeatability); between 0.4 and 0.59 (reasonable repeatability); 0.6 to 0.74 (good repeatability); and 0.75 to 1.0 (excellent repeatability) and this rule of thumb can be used to determine whether the repeatability of the model was enough to delete the effect of sexer from the model, providing a measure of the accuracy of scoring of the sexers, following the guidelines from Fleiss and Cohen [53]. Then, 95% confidence intervals were computed following the expression 95% kappa Confidence Interval (CI) = κ ± 1.96 SEκ, where, SEκ = [(po (1 − po)/n (1 − pe) 2] 0.5, with the procedure of SPSS Statistics Cross tables for Windows, Version 24.0, IBM Corp. (2016) (Armonk, NY, USA). ICC and 95% CI were calculated with the Reliability Analysis routine of the Scale procedure of SPSS Statistics for Windows, Version 24.0, IBM Corp. (2016). Average measures are reported in Supplementary Table S1 as an index for the reliability of the three sexers averaged together in opposition to Single measures, which is an index for the reliability of the ratings for one, typical, single rater [54].

### 2.8. Statistical Analyses

Given one animal cannot be assigned male and female at the same time, there was independence of observations and the dependent variable comprises exclusive and exhaustive categories. Real sex was determined when the chicks were 1.5 months old. Males were coded as 1 and females were coded as 2. Shapiro–Wilk Francia’s tests for normality were performed with StataCorp Stata version 14.2. As normality assumption had been generally violated, a nonparametric approach was followed.

First, the association between the variety of the Utrerana chicken breed (franciscan, white, black, and partridge) and the ability to succeed or fail when assigning sex for the 10 methods was tested using a chi-square independence test. A significance level close to zero means that our variables are very unlikely to be completely unassociated in the population. However, this does not mean the variables are strongly associated; a weak association in a large sample size may also result in *p* = 0.000. Cramér’s V indicates the strength of the association, defined as

$$\phi_{c}=\sqrt{\frac{\chi^{2}}{N\left(k-1\right)}},$$

where $\phi_{c}$ denotes Cramér’s V; *χ*^2^ is the Pearson chi-square statistic, *N* is the sample size involved in the test, and k is the lesser number of categories of either variable [55]. As suggested by Nolan and Heinzen [56], when using Cramér’s V small effect associations range from 0.0 to 0.10, medium effect associations from 0.3 to 0.5, and large effect associations from 0.5 to anything above. The same author would recommend that the interpretation of effect size should consider a statistically significant measure (*p* < 0.05) with a small effect size or greater to indicate a meaningful difference.

Then, medians were compared to determine whether each of the 10 methods were more likely to succeed or fail depending on each variety. Once we identified the methods whose accuracy to assign sexes may be conditioned depending on the variety, we assessed which combinations of methods may be more accurate to predict sex regardless the variety tested. As all the records for method 6 reported the same value for assigned sex (2: female), given the animals’ combs had not developed at any of the moments of evaluation, method 6 was not considered in the following methods aimed at addressing differences. Method 9 was able to assign sex in the franciscan variety more accurately than when the rest of varieties were considered.

To this aim, we studied three different sex assignment criteria cases. The first sex assignment criteria considered the information from bibliography regarding the criteria to follow when determining whether a certain chick is a male or a female proposed by Manuel Llanos Compan [11]. The second assignation criteria was carried out considering the opposite criteria as that reported by bibliography for each of the methods, that is if bibliography interpreted a sign in a certain chick as male determinant, we considered that animal a female and vice versa. The third assignation criteria maximized sex assignation rearranging the criteria obtained from bibliography in a way that the highest number of animals was correctly assigned for each of the assignation criteria.

The McNemar’s test was used to determine if there were differences in the proportion of animals whose sex had been correctly assigned using 10 different methods between two related groups (on the dichotomous dependent variable of real sex; male and female) for the three assignation criteria reported above.

Then, and as a possible overlapping among the predictive power of methods could have occurred, a binary logistic regression was performed to ascertain the effects of the different methods to predictively attribute sex on the likelihood that the sex of the chicks tested was correctly assigned. That is, we assessed how using the different criteria considered in assignation criteria 1, 2, and 3 increased or decreased the predictive power of the methods resulting significant at a binary logistic regression analysis. To this aim, we assessed the effects on the likelihood to correctly assign males in respect to females of changes of one-unit increases (from male to female and vice versa) occurring in the value for sex assigned through significant methods while the rest were kept constant. Binary logistic regression was carried out from the Regression task from SPSS Statistics for Windows, Version 24.0, IBM Corp. (2016).

## 3. Results

ICC and 95% IC aimed at testing for the intersexer reliability across methods, which proved to be highly reliable as there was statistically significant highly good to excellent agreement between the three sexers’ judgements across the 10 methods tested (Supplementary Table S1).

Shapiro Francia’s W’ test showed that assigned sex by the 10 methods in the three assignation criteria, was normally or almost normally distributed (*p* > 0.05). Descriptive statistics are shown in Supplementary Table S3. Eighty-one chicks were submitted to the 11 sex assignation methods and then tested for their real sex. Sex assignation and signs related per each of the 10 methods used in Utrerana hens is shown in Supplementary Table S2.

First, a chi-square independence test showed the variety substantially conditioned the ability to succeed or fail when assigning sex for Method 1.2: Egg width test, Method 2: English test, Method 6: Combs and Method 9: Legs (*p* < 0.05), while it did not condition the results obtained from the other methods. Differences in the medians suggested Method 1.2: Egg width test was more likely to be able to assign franciscan and white animals correctly. Method 2 was more likely to correctly assign sex in white and black animals, by contrast, method 6 was more likely to assign sex correctly in black Utrerana hens. As all the records for method 6 reported the same value for assigned sex (2: female), given the animals’ combs had not developed at any of the moments of evaluation, method 6 was not considered in the following methods aimed at addressing differences (Table 1 and Table 2).

Afterwards, an exact McNemar’s test determined that there was a statistically significant difference in the proportion of animals whose sex had been correctly assigned using each of all the 10 remaining methods except for method 2: egg width test and method 9: legs (*p* > 0.05) for assignation criteria 1 with respect to real sex. Assignation criteria two only reported statistically significant results for methods 2 (English test), 5 (down feathers), 7 (wing fan), and 10 (behavior/coping styles) (*p* < 0.05) compared to real sex. However, for assignation criteria three, results were similar, thus statistically significant (*p* < 0.05), except for method 1.2 (egg width test), method 9 (legs length), and method 4 (tail inclination) compared to real sex (Table 3).

The logistic regression model was statistically significant, χ^2^(10) = 22.974, *p* < 0.001. Given the three assignation criteria comprised the same variables (methods), they all explained 33.1% (Nagelkerke R^2^) of the variance in confirmed sex, presenting a likelihood of correctly classifying 86.7% of males and 61.1% of female cases, resulting in an overall predictive percentage of 75.3%.

When studying assignation criteria 1, the probability of attributing sex correctly occurring based on a one unit change in method 10 (behavior/coping styles), method 4 (cloaca), and method 3 (tail inclination), when all other methods are kept constant was statistically significant (*p* < 0.05) 7.176, 4.235, and 0.156 times greater, respectively, to assign males correctly as opposed to females (Table 4).

When studying assignation criteria 2, the probability of attributing sex correctly occurring based on a one unit change in method 10 (behavior/coping styles), method 3 (tail inclination), and method 4 (cloaca), when all other methods are kept constant was statistically significant (*p* < 0.05) 7.176, 6.420, and 4.235 times greater, respectively, to assign males correctly as opposed to females (Table 5).

When studying assignation criteria 3, the probability of attributing sex correctly occurring based on a one unit change in method 3 (tail inclination), method 4 (cloaca), and method 10 (behavior/coping styles), when all other methods are kept constant was statistically significant (*p* < 0.05) 6.420, 0.236, and 0.139 times greater, respectively, to assign males correctly as opposed to females (Table 6).

## 4. Discussion

The genetic background behind sexual dimorphism comprises the differences between males and females of the same species, such as in color, shape, size, structure, behavior, or cognition that are caused by the inheritance of one or the other sexual pattern in the genetic material [29]. An important finding to have emerged from the chicken studies is that sexual dimorphism at the molecular level can occur well prior to sexual dimorphism at the morphological level. Genes such as DMRT1, ASW, and FET1, for example, all show sexually dimorphic expression as early as day 3.5, well prior to the histological onset of gonadal sex differentiation at day 6.5 [30]. However, the high costs involved in the implementation of molecular techniques makes the development of early sex assignation techniques become especially relevant, even more in the case of local breeds, whose market opportunities are still developing, thus have not reached a sufficient level of profitability as to ensure their survival and satisfy the increasing market demands.

These differences not only attain species but also certain domestic breeds or even varieties [57]. Given the varieties may present different sexual dimorphism patterns, there is a relatively strong possibility that sex determination methods may not be valid across the same varieties of a particular breed. The conditioning effect of the variety on the success to determine sex is supported by the results obtained at the chi-square test of independence and differences in the median, which reflects the significant relationships existing between the categorical sex variable and some of the methods of sexing employed (Table 1 and Table 2).

Egg width, the English test, combs and leg length may present significantly moderately high differences in the suitability to successfully determine sex across the varieties of the Utrerana hen (white, partridge, black, and franciscan). In these cases, a bias may occur towards either of the two sexes as shown by median values shown in Table 2. The rest of tests are appropriate to determine sex for all the individuals, with independence of the variety that they belong to, as results may not be affected by such factor.

When we assessed the results for the differences across the three different assignation criteria, results varied. This finding may rely on the bias occurring due to the particular dimorphic characteristics which may differ across the different varieties (Table 3).

No difference seems to exist across varieties when egg length was compared. However, the egg length test was able to significantly detect differences between females and males when the assignation criteria found in literature (first assignation criteria) was used and when such criteria was reversed (second assignation criteria) (that is if a male was reported in literature to present a certain characteristic while a female did not, we considered the opposite possibility, that is males lacked the characteristic and females presented it).

Contrastingly, egg width reported statistically different results across varieties as shown in Table 1 and Table 2. Table 2 shows that the results for egg width are biased towards incorrectly assigning male sex to chickens in franciscan and white varieties, while they were prone to incorrectly assign females in black and partridge varieties, something that did not occur for egg length (χ^2^ = 2.503, df = 3, *p* = 0.47). Egg width test was able to detect significant differences between females and males when the assignation criteria found in literature (first assignation criteria) was used and when such criteria was reversed (second sex assignation). For the third assignation criteria, such differences were not detected as reported in Table 3.

The relationship between and the influence of egg measurements on the determination of chicken sex has been reported on only a few occasions in literature. Yilmaz-Dikmen and Dikmen [58], reported the effects of egg shape index (*p* = 0.001), egg length (*p* = 0.0018), egg width (*p* < 0.01), and volume (*p* = 0.004) of the egg had a significant effect on the sex of hatching chick of Super Nick White Layers. This could have been expected as the mathematical methods to compute egg shape index or egg volume reported in literature widely depend on egg width and length parameters. In particular, egg volume has been reported to be significantly different between males and females in some avian species [35,58]. For example, using molecular techniques, house sparrow eggs containing male embryos were reported to be significantly larger than those containing female embryos, considering they are laid randomly with respect to laying order [34]. The same authors speculated that this sexual dimorphism of eggs was adaptive, because male house sparrows were more prone to present a greater variability in condition-dependent reproductive success than females. Such results provided further evidence of the ability of females to detect or control ovulation of either male or female ova and to differentially invest in one sex over the other. This was supported by Mead, Morton, and Fish [35] with male eggs in Mountain White-crowned Sparrows (*Zonotrichiu leucophrys oriantha*) being highly significantly slightly larger than those of female eggs for each of the five consecutive years that the study lasted (*p* < 0.01).

Some authors have suggested plumage variety may play an important role. In particular, the greater or lesser accuracy when determining the sex of an individual have been reported to potentially be conditioned by the variety of hen Utrerana for method 1.2 (width of the egg), method 2 (English method), method 6 (degree of development of the crest), and method 9 (leg length). In the particular case of method 6, this would not provide conclusive data for any variety if the tasks of sex assignment are developed before the 1.5 month age, as it happens in our case. Hence, this method was discarded as a method of sexing for subsequent analyses, and it was rather used to confirm sex when the animals reached 1.5 months of age (6 weeks).

For Brown Leghorn, adult plumage is a sexually dimorphic feature, in terms of the kind and distribution of color in the individual feathers, and in one or more of the seven areas in which plumage color differences may distinguish among breeds and varieties. Furthermore, males are characterized by the structure of the feathers of the neck and saddle hackles, and by the presence of the large tail sickles in males. In other cases, such as the White Leghorn, the sexes are to be distinguished only by the structural differences in the hackle feathers and by the large tail sickles of the male. In the case of certain other breeds, the Campines and the Sebrights, for example, the plumage of the male is identical with that of the female both in coloration and in structure. Cocky-feathering in the case of such varieties as the Brown and the White Leghorns can be regarded as a trustworthy indication that within the body there is, or was at the time when the plumage was developed, active functional testicular tissue; henny-feathering as an indication that there is, or was when the plumage was developed, active functional ovarian tissue.

The underlying causes and the likely physiological consequences of the association of certain genes with sex biased expression were not the focus of this study. However, these remain interesting topics for future research. For instance, sex-biased gene expression of various steroid hormones in adrenals may potentially contribute to the extensive sexually dimorphic behavioral and physiological traits observed in chickens [42]. Chronic [59,60] and acute [55] stress may cause immediate, short- and long-term changes in physiology, behavior, and gene regulation. These responses may vary between individuals as well as between classes of individuals within a single species (for example, between sexes of animals with different coping styles) [58,61] and their characterization may greatly increase our understanding of stress effects in general and their differential extent between males and females [54].

Stress causes cascades of both immediate and long-term changes in physiology, behavior, and gene regulation. However, both short- and long-term responses may vary between individuals as well as between classes of individuals within a single species (for example, between sexes, or between animals with different coping styles) [61]. Characterization of such differences may greatly increase our understanding of stress effects in general. Chronic stress in chickens (*Gallus gallus domesticus*) has been reported to be the case, for example, changes in learning ability, social dominance, feeding behavior, and gene expression [59,60], and the extent of these effects were found to differ between the sexes [62]. Whether similar sex differences are also found in response to acute stress experiences (i.e., stressors acting over short periods) is less well understood [63].

Contextually, the study by Zappia and Rogers [64] examined the effect of testosterone on the asymmetry of visual discrimination performance of young chicks. Two-week-old chicks were tested on the pebble floor visual discrimination task. Male chicks were found to have brain asymmetry for visual discrimination learning, since chicks tested binocularly, or tested monocularly using their right eye system, have superior learning performance compared to chicks tested monocularly using their left eye system.

Among zoometric parameters, neonatal tail posture is a sexually dimorphic behavior with females more biased leftwards than males. Prenatal exposure of female chickens to testosterone propionate (TP) but not dihydrotestosterone propionate (DHTP) shifts the population pattern of tail posture to the right. Contrastingly, no effect was found with male pups [65].

Casual observations suggested that the difference in size between male and female fowls is more marked in the tarso-metatarsus than in other bones of the appendicular skeleton. Plumage usually refers to the shape, size, and appearance of the feathers on a fowl at any specific time. Plumage, therefore, continually changes in juvenile individuals, and becomes fairly consistent in adults. Neck feathers are usually referred to as the “hackles”. The hackle feathers of the adult male are very distinctive and can alone be used to differentiate sex of the chicken. Male hackles are long, pointed, and usually reach down to the wings, while in the female the hackles are less distinctive, rounded in shape, and usually blend in with the other body feathers. The hackle feathers of many-colored males are greatly prized for producing “flies” for fishermen.

The plumage of most adult males and females also differ in the pelvic region, where again, the males have much longer and more pointed tail coverts. Abnormalities in growth of these feathers, or physical damage, can cause disruption of this normal arrangement of the primaries, often leading to a characteristic ruffling or “sticking-out” of one or two feathers. In broiler chickens, this latter condition is sometimes referred to as “helicopter wing”, where one or two primaries on each side of the body stick out at a 25–45° angle compared to their normal plane, which is parallel to the body [37].

Thyroid hormone has been reported to be associated to much darker black/brown color due to extra melanin deposition in eggshells. Sex hormones also influence feather color. For example, in Brown Leghorn fowls’ sexual dimorphism of feather color is under the control of estrogen although the effect is influenced by thyroid status [66,67]. Sex hormones can (but do not always) influence melanoblast differentiation. Genetics rather than hormone balance essentially control the different feather down colors of day-old chicks. However, if estrogen or testosterone levels are altered in the developing embryo, down color can be affected. The authors of [68] suggest that selection for genes that suppress feather color results in improved feed efficiency in layers, possibly due to better retention of feather cover as fowls age.

The conditioning effect of variety on the accuracy of sexing methods becomes evident by the results obtained at the χ^2^ independence test and the differences in the medians, which suggest the existing significant relationship between the dicotomic variable of sex and some of the sexing methods employed. In particular, the greater or lesser precision in determining an individual’s sex could depend on the variety of Utrerana hen for method 1.2 (egg length), method 2 (English method), method 6 (degree of development of the crest), and method 9 (length of legs). In the particular case of method 6, this method would not report relevant information as sex assignment tasks are carried out before the age of 1 month, as is our case, the reason why this method was discarded in subsequent analyses, but used for sex confirmation.

Once the influence of the variety of the Utrerana breed on sexing methods in chickens at an early age has been determined, the best accurately performing combination of methods was determined. Given their efficiency regardless of the variety of the individuals, assignation criteria 2 reported the most accurate results when the gender allocation criteria used was the one reversing the information normally provided by literature. Hence, as opposite to literature, if the chick kicks, it will be considered a male; while if the animal remains motionless, it will be considered a female.

The proportion of animals whose sex had been correctly assigned differed significantly between methods, namely, for assignation criteria 1, all methods used except method 1.2 (egg width) and method 9 (leg length), allowed for certain sexing at a statistically significant level. However, in assignation criteria 2, only method 3 (English method), method 5 (general down feathers coloration), method 7 (wing fan), and method 10 (behavior/coping styles) reported these same significant results. In the case of assignation criteria 3, the results are similar to those obtained for assignation criteria 1, with no statistically significant proportion of animals sexed for methods 1.2 (egg width), 3 (tail inclination), and 9 (legs length).

Finally, the percentage of variance explained (75%) in the sex of chickens at an early age is the same for the three assignation criteria considered. This could have been expected as only sex proportions in the population was considered and the same within sample variability occurred. However, although the explanatory power of all assignation criteria is identical, that is they presented an equal potential to explanation of variance, assignation criteria 2 resulted to be the most efficient as it correctly assigns males more frequently.

## 5. Conclusions

Conclusively, the methodology used until now has been useful for commercial poultry lines with a short life cycle in which the early determination of the sex of individuals aims to remove from the production cycle that category of sex that is not of interest to the specific productive objective pursued. However, in the case of poultry breeds with other uses and husbandry/farming systems, as well as presenting large differences regarding production data (meat or eggs), these findings could evidence the prevailing need to adapt the methods of determining sex to each breed and/or avian genetic lineage, as morphological (degree of comb development and leg length), ethological (English method), and productive (egg width) characteristics could comprise an efficient tool to tailor specific sexing tasks relying on the basis of a specific phenotypic differentiation of each breed. Characterizing differences across varieties may be of help when assessing the results of crossbreeding and selection plans when seeking the obtention of individuals displaying certain desired characteristics for the production objective. In any case, it is advisable to have a record of the battery of tests or methods used, the predictive potential of the same and the results obtained, to ultimately apply the appropriate corrections and adaptations in each situation with the aim to achieve as accurate and reliable a determination of the sex relationship in a population as possible, an action of particular importance in the implementation and improvement of conservation and breeding programs for endangered species.

## Figures and Tables

**Table 1:** Summary of the results for strength of association between the variety of the Utrerana chicken breed and the ability to succeed or fail when assigning sex for the 10 methods using chi-square independence test and Cramér’s V.

Method	Chi-Square	df	Asymp. Sig.	Cramér’s V
Method 1.1: Egg length test	2.503	3	0.47	N/A
Method 1.2: Egg width test	8.042	3	0.04	0.317
Method 2: English test	7.736	3	0.05	0.311
Method 3: Tail inclination	0.918	3	0.82	N/A
Method 4: Cloaca	0.944	3	0.81	N/A
Method 5: Down feathers	6.269	3	0.10	N/A
Method 6: Combs	10.685	3	0.01	0.365
Method 7: Wing fan	6.382	3	0.09	N/A
Method 8: Head size and morphology	4.387	3	0.22	N/A
Method 9: Legs	7.974	3	0.05	0.316
Method 10: Behavior/Coping styles	7.403	3	0.06	N/A

N/A: It does not apply to compute Cramér’s V as chi-square did not report a significant association.

**Table 2:** Median value for sex assignation (1: male; 2: female) for each of the 10 methods used sorted by variety of Utrerana hen breed.

Method	Franciscan	Partridge	White	Black
Method 1.1: Egg length test	2	2	2	2
Method 1.2: Egg width test	1	2	1	2
Method 2: English test	2	2	1	1
Method 3: Tail inclination	1	1	1	2
Method 4: Cloaca	2	1	1	1
Method 5: Down feathers	1	2	1	2
Method 6: Combs	2	2	2	1
Method 7: Wing fan	2	2	2	1
Method 8: Head size and morphology	2	2	1	1
Method 9: Legs	1	2	2	2
Method 10: Behavior/Coping styles	1	1	1	2

**Table 3:** Summary of the results for McNemar’s test used to determine if there were differences in the dichotomous dependent variable of sex assigned using 10 different methods between two related groups (real sex; male and female) for the three assignation criteria possibilities in Utrerana chicks.

Method	Assignation Criteria 1 (Literature)		Assignation Criteria 2 (Opposite to What is in Literature)		Assignation Criteria 3 (Combination of Best Performing Criteria)	
	Chi-Squared	Asymptotic Significance	Chi-Squared	Asymptotic Significance	Chi-Squared	Asymptotic Significance
Method 1.1: Egg length test	4.356	0.04	4.356	0.04	0.694	0.40
Method 1.2: Egg width test	0.089	0.77	0.089	0.77	0.694	0.40
Method 2: English test	35.220	0.00	35.220	0.00	21.025	0.00
Method 3: Tail inclination	3.698	0.05	0.893	0.34	0.893	0.34
Method 4: Cloaca	11.605	0.01	11.605	0.01	3.349	0.07
Method 5: Down feathers	10.256	0.01	10.256	0.01	20.024	0.00
Method 7: Wing fan	25.689	0.00	25.689	0.00	17.361	0.00
Method 8: Head size and morphology	3.841	0.05	3.841	0.05	0.432	0.51
Method 9: Legs	2.132	0.14	2.132	0.14	0.000	1.00
Method 10: Behavior/Coping styles	12.033	0.01	12.033	0.01	15.373	0.00

**Table 4:** Probabilities of attributing sex correctly occurring based on a one-unit change in any of the methods when all other independent variables are kept constant for assignation criteria 1 in Utrerana chicks.

Method	B	S.E.	Wald	df	Significance	Exp(B)	95% C.I. for Exp(B)
Method 1.1: Egg length test	0.226	0.626	0.130	1	0.72	1.253	0.367–4.274
Method 1.2: Egg width test	−0.923	0.616	2.241	1	0.13	0.397	0.119–1.330
Method 2: English test	1.289	1.353	0.908	1	0.34	3.630	0.256–51.458
Method 3: Tail inclination	−1.859	0.650	8.176	1	0.01	0.156	0.044–0.557
Method 4: Cloaca	1.443	0.747	3.737	1	0.05	4.235	0.980-18.301
Method 5: Down feathers	−0.431	0.703	0.375	1	0.54	0.650	0.164–2.580
Method 7: Wing fan	−0.651	1.031	0.398	1	0.53	0.522	0.069–3.933
Method 8: Head size and morphology	0.194	0.583	0.110	1	0.74	1.214	0.387–3.805
Method 9: Legs	0.851	0.576	2.186	1	0.14	2.342	0.758–7.237
Method 10: Behavior/Coping styles	1.971	0.787	6.266	1	0.01	7.176	1.534–33.579
Constant	−1.338	1.757	0.580	1	0.45	0.262	

**Table 5:** Probabilities of attributing sex correctly occurring based on a one-unit change in any of the methods when all other independent variables are kept constant for assignation criteria 2 in Utrerana chicks.

Method	B	S.E.	Wald	df	Significance	Exp(B)	95% C.I. for Exp(B)
Method 1.1: Egg length test	−0.226	0.626	0.130	1	0.719	0.798	0.234–2.722
Method 1.2: Egg width test	0.923	0.616	2.241	1	0.134	2.516	0.752–8.420
Method 2: English test	−1.289	1.353	0.908	1	0.341	0.276	0.019–3.906
Method 3: Tail inclination	1.859	0.650	8.176	1	0.004	6.420	1.795–22.961
Method 4: Cloaca	−1.443	0.747	3.737	1	0.053	0.236	0.055–1.020
Method 5: Down feathers	0.431	0.703	0.375	1	0.540	1.538	0.388–6.104
Method 7: Wing fan	0.651	1.031	0.398	1	0.528	1.917	0.254–14.445
Method 8: Head size and morphology	−0.194	0.583	0.110	1	0.740	0.824	0.263–2.583
Method 9: Legs	−0.851	0.576	2.186	1	0.139	0.427	0.138–1.319
Method 10: Behavior/Coping styles	−1.971	0.787	6.266	1	0.012	0.139	0.030–0.652
Constant	0.772	0.982	0.618	1	0.432	2.164	

**Table 6:** Probabilities of attributing sex correctly occurring based on a one-unit change in any of the methods when all other independent variables are kept constant for assignation criteria 3 in Utrerana chicks.

Method	B	S.E.	Wald	df	Significance	Exp(B)	95% C.I. for Exp(B)
Method 1: Egg length test	0.226	0.626	0.130	1	0.71	1.253	0.367–4.274
Method 1.2: Egg width test	−0.923	0.616	2.241	1	0.13	0.397	0.119–1.330
Method 2: English test	1.289	1.353	0.908	1	0.34	3.630	0.256–51.458
Method 3: Tail inclination	1.859	0.650	8.176	1	0.01	6.420	1.795–22.961
Method 4: Cloaca	1.443	0.747	3.737	1	0.05	4.235	0.980–18.301
Method 5: Down feathers	−0.431	0.703	0.375	1	0.54	0.650	0.164–2.580
Method 7: Wing fan	−0.651	1.031	0.398	1	0.53	0.522	0.069–3.933
Method 8: Head size and morphology	0.194	0.583	0.110	1	0.74	1.214	0.387–3.805
Method 9: Legs	0.851	0.576	2.186	1	0.14	2.342	0.758–7.237
Method 10: Behavior/Coping styles	1.971	0.787	6.266	1	0.01	7.176	1.534–33.579
Constant	−9.027	4.568	3.906	1	0.05	0	

## Supplementary Materials

The following are available online at https://www.mdpi.com/2076-2615/9/12/1165/s1, Table S1. Cronbach’s Alpha, Cohen’s kappa Intra Class Correlation Coefficient, and 95% Confidence interval to test for intersexer reliability testing absolute agreement and consistency across sexing methods; Table S2. Sex assignation and signs related per each of the 11 methods used in Utrerana hens found in literature; Table S3. Descriptive statistics for sex attribution methods in Utrerana chicks.
