# Supplementary material for: Sexual Dimorphism for Coping Styles Complements Traditional Methods for Sex Determination in a Multivariety Endangered Hen Breed
Source: Animals (Basel). 2019 Dec 17;9(12):1165. doi: 10.3390/ani9121165 (PMC6941311; doi:10.3390/ani9121165)
Supplement: Supplementary file 1 [file animals-09-01165-s001.pdf]

animals

Sexual Dimorphism for Coping Styles Complements Traditional Methods for Sex Determination in a Multivariety Endangered Hen Breed

Carlos Iglesias Pastrana [1], Francisco Javier Navas González [1,*], Carmen Marín Navas [1], Ander Arando Arbulu [1], Antonio González Ariza [1], José Manuel León Jurado [2], María Gabriela Pizarro Inostroza [3] and Maria Esperanza Camacho Vallejo [4]

Table S1. Cronbach's Alpha, Cohen's kappa Intra Class Correlation Coefficient and 95% Confidence interval to test for intersexer reliability testing absolute agreement and consistency across sexing methods.

	Sexing Method	Intraclass Correlation	95% Confidence Interval	F Test with True Value 1	df1	df2	Sig	Repeatability Verdict
Absolute agreement	Method 1.1: Egg length test	0.708	0.469–0.850	3.472	28	56	0	Good
	Method 1.2: Egg width test	0.860	0.741–0.929	7.122	28	56	0	Excellent
	Method 2: English test	0.796	0.623–0.897	4.874	28	56	0	Excellent
	Method 3: Tail inclination	0.875	0.769–0.937	8.286	28	56	0	Good
	Method 4: Cloaca	0.671	0.394–0.835	3.044	28	56	0	Good
	Method 5: Side feathers	0.635	0.340–0.814	2.918	28	56	0	Good
	Method 6: Combs	0.775	0.582–0.887	4.346	28	56	0	Excellent
	Method 7: Wing fan	0.643	0.351–0.818	2.911	28	56	0	Good
	Method 8: Head size and morphology	0.752	0.538–0.876	3.943	28	56	0	Excellent
	Method 9: Legs length	0.860	0.741–0.929	7.122	28	56	0	Excellent
	Method 10: Behaviour/Coping style	0.734	0.512–0.865	3.866	28	56	0	Good

Consistency	Method 1.1: Egg length test	0.712	0.472–0.853	3.472	28	56	0	Good
	Method 1.2: Egg width test	0.860	0.740–0.929	7.122	28	56	0	Excellent
	Method 2: English test	0.795	0.620–0.897	4.874	28	56	0	Excellent
	Method 3: Tail inclination	0.879	0.776–0.939	8.286	28	56	0	Excellent
	Method 4: Cloaca	0.671	0.391–0.835	3.044	28	56	0	Good
	Method 5: Side feathers	0.657	0.365–0.828	2.918	28	56	0	Good
	Method 6: Combs	0.782	0.596–0.890	4.587	28	56	0	Excellent
	Method 7: Wing fan	0.770	0.574–0.884	4.346	28	56	0	Excellent
	Method 8: Head size and morphology	0.656	0.364–0.827	2.911	28	56	0	Good
	Method 9: Legs length	0.746	0.530–0.873	3.943	28	56	0	Excellent
	Method 10: Behaviour/Coping style	0.741	0.521–0.870	3.866	28	56	0	Excellent

Table S2. Descriptive statistics for sex attribution methods in Utrerana chicks.

	**Range**	**Minimum**	**Maximum**	**Mean**	**Std. Error**	**Std. Deviation**	**Variance**	**Skewness**	**Std. Error**	**Kurtosis**	**Std. Error**
Method 1.1: Egg length test	1	1	2	1.63	0.054	0.486	0.236	-0.547	0.267	-1.744	0.529
Method 1.2: Egg width test	1	1	2	1.48	0.056	0.503	0.253	0.076	0.267	-2.045	0.529
Method 2: English test	1	1	2	1.93	0.029	0.264	0.069	-3.314	0.267	9.212	0.529
Method 3: Tail inclination	1	1	2	1.37	0.054	0.486	0.236	0.547	0.267	-1.744	0.529
Method 4: Cloaca	1	1	2	1.72	0.05	0.454	0.206	-0.976	0.267	-1.074	0.529
Method 5: Side feathers	1	1	2	1.19	0.043	0.391	0.153	1.652	0.267	0.746	0.529
Method 7: Wing fan	1	1	2	1.88	0.037	0.331	0.11	-2.333	0.267	3.528	0.529
Method 8: Head size and morphology	1	1	2	1.62	0.054	0.489	0.239	-0.492	0.267	-1.803	0.529
Method 9: Leg length	1	1	2	1.57	0.055	0.498	0.248	-0.279	0.267	-1.971	0.529

Method 10: Behaviour/Coping style	1	1	2	1.2	0.045	0.401	0.16	1.548	0.267	0.406	0.529
Confirmed Sex	1	1	2	1.44	0.056	0.5	0.25	0.228	0.267	-1.998	0.529

SKEWNESS	
	If skewness is less than −1 or greater than +1, the distribution is highly skewed.
	If skewness is between −1 and −½ or between +½ and +1, the distribution is moderately skewed.
	If skewness is between −½ and +½, the distribution is approximately symmetric.
Data that are skewed to the right have a long tail that extends to the right, that is a positive Skewness statistic value. In this situation, the mean and the median are both greater than the mode. As a general rule, most of the time for data skewed to the right, the mean will be greater than the median. The situation reverses itself when we deal with data skewed to the left. Data that are skewed to the left have a long tail that extends to the left, that is negatively skewed. In this situation, the mean and the median are both less than the mode. As a general rule, most of the time for data skewed to the left, the mean will be less than the median.	

KURTOSIS	
	A normal distribution has kurtosis exactly 3 (excess kurtosis exactly 0). Any distribution with kurtosis ≈3 (excess ≈0) is called mesokurtic.
	A distribution with kurtosis <3 (excess kurtosis <0) is called platykurtic. Compared to a normal distribution, its central peak is lower and broader, and its tails are shorter and thinner.
	A distribution with kurtosis >3 (excess kurtosis >0) is called leptokurtic. Compared to a normal distribution, its central peak is higher and sharper, and its tails are longer and fatter.

Table S3. Sex assignation and signs related per each of the eleven methods used in Utrerana hens found in Literature.

**Method**	**♂=1**	**♀=2**
Method 1.1: Egg length test	*Dull*	*Long*
Method 1.2: Egg width test	*Wide*	*Pointy*
Method 2: English test	*Does not kick*	*Kicks*
Method 3: Tail inclination	*Straight*	*Downwards*
Method 4: Cloaca	*One protuberance*	*Two protuberances*
Method 5: Down feathers	*Homogeneous*	*Heterogeneous*
Method 6: Combs	*Well-developed*	*Poorly-developed*
Method 7: Wing fan	*Incomplete and unequal*	*Complete and equal*
Method 8: Head size and morphology	*Small and round*	*Large and angled*
Method 9: Legs length	*Long*	*Short*
Method 10: Behaviour/Coping style	*Freezes*	*Flees*
